# Estimating and visualising the trade-off between benefits and harms on multiple clinical outcomes in network meta-analysis

**DOI:** 10.1186/s13643-023-02376-1

**Published:** 2023-11-11

**Authors:** Virginia Chiocchia, Toshi A. Furukawa, Johannes Schneider-Thoma, Spyridon Siafis, Andrea Cipriani, Stefan Leucht, Georgia Salanti

**Affiliations:** 1 Institute of Social and Preventive Medicine, University of Bern, Bern, Switzerland; 2 Graduate School of Health Sciences, University of Bern, Bern, Switzerland; 3 Department of Health Promotion and Human Behavior, Kyoto University Graduate School of Medicine/School of Public Health, Kyoto, Japan; 4 Department of Psychiatry and Psychotherapy, School of Medicine, Technical University of Munich, Munich, Germany; 5 Department of Psychiatry, University of Oxford, Oxford, UK; 6 Oxford Health NHS Foundation Trust, Warneford Hospital, Oxford, UK; 7 Oxford Precision Psychiatry Lab, Oxford Health Biomedical Research Centre, Oxford, UK

**Keywords:** Network meta-analysis, Treatment performance, Multiple outcomes, Benefits, Harms, Trade-off, Spie charts

## Abstract

**Background:**

The relative treatment effects estimated from network meta-analysis can be employed to rank treatments from the most preferable to the least preferable option. These treatment hierarchies are typically based on ranking metrics calculated from a single outcome. Some approaches have been proposed in the literature to account for multiple outcomes and individual preferences, such as the coverage area inside a spie chart, that, however, does not account for a trade-off between efficacy and safety outcomes.

We present the net-benefit standardised area within a spie chart, $SAWIS$ to explore the changes in treatment performance with different trade-offs between benefits and harms, according to a particular set of preferences.

**Methods:**

We combine the standardised areas within spie charts for efficacy and safety/acceptability outcomes with a value *λ* specifying the trade-off between benefits and harms. We derive absolute probabilities and convert outcomes on a scale between 0 and 1 for inclusion in the spie chart.

**Results:**

We illustrate how the treatments in three published network meta-analyses perform as the trade-off *λ* varies. The decrease of the $SAWIS$ quantity appears more pronounced for some drugs, e.g. haloperidol. Changes in treatment performance seem more frequent when SUCRA is employed as outcome measures in the spie charts.

**Conclusions:**

$SAWIS$ should not be interpreted as a ranking metric but it is a simple approach that could help identify which treatment is preferable when multiple outcomes are of interest and trading-off between benefits and harms is important.

**Supplementary Information:**

The online version contains supplementary material available at 10.1186/s13643-023-02376-1.

## Background

When multiple competing treatments are available for a specific condition, network meta-analysis (NMA) is used to identify which one is preferable by estimating relative treatment effects between each pair of treatments for a given outcome of interest [1, 2]. From these relative treatment effects, one can obtain summary statistics to measure the performance of an intervention. Such quantitative measures are called ranking metrics and are used to produce treatment hierarchies from the most preferable to the least preferable option. Among the most commonly reported treatment hierarchies we find those based on the probability of producing the best value, the SUCRA or its frequentist equivalent, the P-score, as well as those produced by ranking the relative treatment effects against a reference or control treatment (e.g. placebo) [3–5]. These ranking metrics are typically calculated for a single outcome, so network meta-analyses often present several treatment hierarchies for all harmful and beneficial outcomes. Extensions of the existing ranking metrics that involve multiple outcomes have been recently presented. Mavridis et al. extended the idea of the P-score for multiple outcomes [6] and Daly et al. introduced a new framework, the spie charts, to measure the effectiveness or safety of each treatment on multiple outcomes [7]. In a spie chart, the importance of each outcome is represented by the angle of an outcome-specific sector composing the spie chart, whose coverage area represents the quantity by which to rank the treatments. Efficacy and safety outcomes should not, however, be plotted on the same spie chart since a single value for the resulting area inside could mask important information on safety, so the authors suggest producing two separate spie charts, one for benefit and one for harmful outcomes.

The aim of this paper is to combine the areas of the two spie charts to produce a visual and numerical way to explore the sensitivity of treatment performance to the different perceptions of the trade-off between the benefit and harms of treatments, subject to a particular set of preferences in terms of outcome importance. We illustrate if and how the trade-off between benefits and harms on multiple clinical outcomes varies for the treatments of three published network meta-analyses.

## Motivating examples

The first example is a network of head-to-head studies investigating 18 antidepressants for the acute treatment of adults with major depressive disorder [8]. We consider two efficacy dichotomous outcomes: response to treatment (defined as a reduction of at least 50% in the score between baseline and week 8 on a standardised rating scale for depression) and remission. We also consider two outcomes about harms: acceptability (dropout due to any cause) and tolerability (dropout due to adverse events). The presentation of both beneficial and harmful outcomes is important for the clinical decision-making process, because some antidepressants, like amitriptyline, have a good efficacy profile, particularly in terms of response to treatment, but perform poorly in terms of acceptability and tolerability.

The second example is a network of placebo-controlled studies of antipsychotics for the acute treatment of adults with multi-episode schizophrenia [9]. We consider one efficacy outcome, overall symptoms of schizophrenia as measured by rating scales, and four safety outcomes: use of antiparkinson medication (as a proxy of extra-pyramidal symptoms), weight gain, prolactin elevation, and QTc prolongation (as a proxy of cardiac risk). Some antipsychotics show a clear distinction between their own efficacy and safety profiles. For instance, haloperidol and olanzapine are among the most effective antipsychotics but they are associated with high rates of antiparkinson medication use and weight gain, respectively. The chosen outcomes are not available for all drugs, so we include only 14 antipsychotics plus placebo in the application of our proposed approach.

The third example is a network of pharmacological and dietary‑supplement treatments for autism spectrum disorder [10]. We consider two efficacy outcomes: changes in core symptoms for social-communication difficulties and repetitive behaviours as measured by any validated scale, and a safety outcome, i.e. number of patients reporting any adverse event. Due to missing outcomes for some of the treatments, the implementation of our approach includes only placebo and 19 treatments.

The network graphs of the three motivating examples, as reported in the original publications, are available in Figures A–C of Additional file 1.

## Methods

We first introduce the standardised area within a spie chart as reported by Daly et al. [7] and then we illustrate how we combine it with a trade-off value. Let us consider *j* = *1, …, J* outcomes and *i* = *1, …, N* treatments. The measures ${y}_{ij}$ for a treatment *i* and an outcome *j* range between 0 and 1 and have weights ${w}_{j}$ reflecting the importance of outcome *j*.

### The standardised area within a spie chart (A^std^)  

For a specific treatment *i*, the formula for the standardised area within a spie chart (${A}_{i}^{std}$) for $J$ outcomes measures ${y}_{ij}$ with weights ${w}_{j}$ is the following:

1 $$\begin{array}{c}A_i^{std}=\frac1{2\pi}\sum\limits_{j=1}^Jw_jy_{ij}^2\end{array}$$

The weights ${w}_{j}$ represent the angles of the *J* sectors composing the spie chart and range between 0 and 2π, where ${w}_{j}=0$ implies outcome *j* does not contribute to the area, i.e. it is irrelevant for the purpose of ranking the treatments, and ${w}_{j}=2\pi$ implies outcome *j* is the sole contributor to the area, i.e. it is the only outcome considered important to rank the treatments. Daly et al. illustrate various methods to derive the contribution of the outcomes in terms of weights. These include but are not limited to preference elicitation from decision-makers or experts, coefficients from prognostic models, and more generally, evidence in the literature [7]. The efficacy and safety spie charts for the included treatments and chosen outcomes of the three motivating examples are reported in Figures A–F of Additional file 2.

To be plotted on the same spie chart, the measures ${y}_{ij}$ for the *J* outcomes must be on the same bounded scale, such as SUCRA or an absolute probability. However, this is challenging, as often one may want to include a combination of dichotomous, continuous, or time-to-event outcomes. In Additional file 3, we describe some existing methods to convert treatment effects of dichotomous and continuous outcomes to ${y}_{i}$ scaled and bounded between 0 and 1 [11–14]. When it is not possible to perform these conversion methods and/or obtain absolute probabilities, the alternative of using SUCRA as the outcome measures ${y}_{i}$ can always be employed, as shown by Daly et al. [7]. Another important aspect to note is that the chosen outcomes to be included in the same spie chart must also be measured in the same direction. That means, that higher values for the efficacy outcomes indicate a higher benefit, while higher values for the safety outcomes indicate a higher harm. Therefore, to outperform its competitors, a treatment would achieve the largest area within the efficacy spie chart but the smallest area within the safety spie chart. Another issue with the spie chart method is that it is unlikely to have all chosen outcomes available for all treatments. The authors do not provide a specific or preferred solution to this, leaving the choice of how to deal with this missingness to the user. To illustrate our proposed method, we only include in the spie charts those treatments that have data available for all chosen outcomes.

### Benefit and harms trade-off: the net-benefit standardised area within a spie chart (SAWIS)

To trade off between benefits and harms, we introduce a value *λ* to combine the standardised areas within the spie charts for efficacy and safety/acceptability outcomes within the same formula. The latter will produce a numerical quantity, the net-benefit standardised area within a spie chart ($SAWIS$) that could be interpreted as the “net benefit” with the treatment, measured on a SAWIS difference scale

2 $$\begin{array}{c}{SAWIS}_{i}={A}_{i}^{B}-\lambda *{A}_{i}^{H}\end{array}$$

${A}_{i}^{B}$ is the (standardised) area within the spie chart for benefit from efficacy outcomes, while ${A}_{i}^{H}$ is the (standardised) area within the spie chart for “harm”, that includes safety and acceptability outcomes, as defined in Eq. (1). We set $\lambda ={}^{1}\!\left/ \!{}_{u}\right.$, where *u* can vary between 1 and infinity to reflect the amount of harm that we are willing to accept for an increase in benefit, measured on the SAWIS scale.

${SAWIS}_{i}$ cannot easily be interpreted as a ranking metric. In our case, the coverage area within a spie chart is a weighted sum of the efficacy or safety and acceptability outcomes measured on a 0–1 scale, and the obtained value is a difference adjusted for a specific willingness-to-pay in terms of negative outcomes ($\lambda$). In addition, it might be difficult to elicit plausible values for *u*, as the unit of measure is not a probability, change in scores, or a specific outcome that the patients would be able to trade-off with harms, but the area inside a spie chart. Therefore, our approach should be employed as a sensitivity analysis to show which treatment is preferable for different trade-offs between benefits and harms, i.e. for the whole range of $\lambda$ values.

## Implementation

We developed an R function to implement the methods described above, incorporating the Spie chart R code provided by its authors. The user must specify the efficacy outcomes and safety/acceptability outcomes as separate vectors and, similarly, the relative vectors of weights as values between 0 and 1. The weight values must add up to 1 for the efficacy and safety/acceptability outcomes separately. The user is also required to specify outcome labels as string vectors for the two sets of outcomes separately and a value, or a series of values, for the trade-off *λ*. As a default, the function calculates the quantity, ${SAWIS}_{i}$, for *λ* ranging from 0 to 1 by increment of 0.05. The function returns three objects: the benefit spie charts and the harms spie charts, both containing the plot and the value of the area within the spie chart for each treatment; and a table showing the ${SAWIS}_{i}$ values for the treatments at each value of *λ*. The R codes for the function and to reproduce the plots in this paper are freely available on GitHub (https://github.com/esm-ispm-unibe-ch/nb-spie).

## Results

### Ranking antidepressants 

We present the ${SAWIS}_{i}$ for the network of 18 antidepressants for the acute treatment of adults with major depressive disorder. We show how treatment performance varies for different values of the trade-off $\lambda$.

We first estimated for each treatment the absolute probabilities ${y}_{i}$ of response or risk for the outcomes as described in Equations (1) and (2) of Additional file 3. Fluoxetine was chosen as the reference drug, so we estimated the odds in this control group by meta-analysing the Fluoxetine arms.

The probability ${p}_{\mathrm{Fluoxetine}}$ for response, remission, dropouts for any cause, and due to side effects were 0.569, 0.347, 0.236, and 0.078, respectively, as reported in Table 1.

**Table 1:** Probabilities of response to treament, remission, dropout due to any cause, and dropout due to side effects, estimated from the network of 18 antidepressants for the acute treatment of adults with major depressive disorder

	**Response**	**Remission**	**Dropout for any cause**	**Dropout for side effects**
Agomelatine	0.613	0.362	0.208	0.054
Amitriptyline	0.621	0.381	0.268	0.120
Bupropion	0.645	0.459	0.249	0.095
Citalopram	0.583	0.333	0.228	0.074
Clomipramine	0.569	0.378	0.315	0.171
Duloxetine	0.601	0.383	0.295	0.150
Escitalopram	0.638	0.393	0.212	0.066
Fluoxetine	0.569	0.347	0.236	0.078
Fluvoxamine	0.569	0.365	0.276	0.098
Milnacipran	0.596	0.352	0.249	0.070
Mirtazapine	0.629	0.371	0.246	0.096
Nefazodone	0.579	0.342	0.273	0.117
Paroxetine	0.611	0.375	0.244	0.092
Reboxetine	0.524	0.295	0.329	0.162
Sertraline	0.596	0.357	0.234	0.067
Trazodone	0.540	0.340	0.278	0.109
Venlafaxine	0.610	0.377	0.262	0.124
Vortioxetine	0.682	0.413	0.176	0.062

After consulting with clinicians, we gave a weight of 0.3 and 0.7 to the response and remission outcomes respectively; and weights of 0.7 and 0.3 to dropout due to side effects and dropout due to any cause outcomes, respectively.

**Fig. 1:**
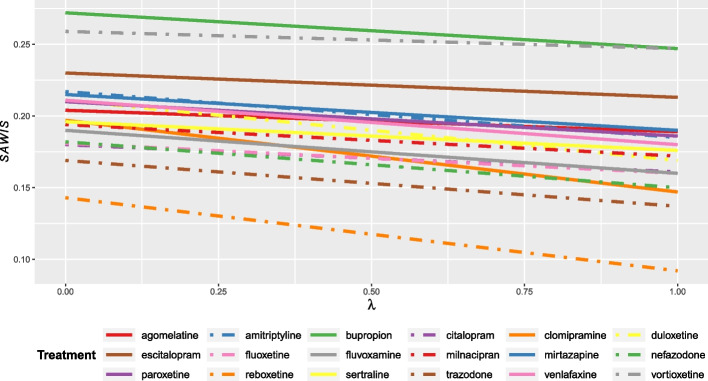
${SAWIS}_{i}$ for the network of 18 antidepressants for the acute treatment of major depressive disorder. The benefit spie chart included response and remission with weights 0.3 and 0.7, respectively, and the harm spie chart included dropout due to side effects and due to any cause with weights 0.7 and 0.3, respectively

The ${SAWIS}_{i}$ values for different $\lambda$ are shown in Additional file 4 and illustrated in Fig. 1. In line with Eq. (2), the ${SAWIS}_{i}$ values decrease with increasing values of $\lambda$. However, this decrease is less pronounced for some treatments, such as vortioxetine which, for the chosen weights, seems to retain its high performance for any trade-off between beneficial and harmful outcomes. Whatever the trade-off between benefits and risks, reboxetine remains the worst-performing drug, while vortioxetine, bupropion, and escitalopram are consistently the best options.

### Ranking antipsychotics 

To transform the efficacy outcome, overall symptoms of schizophrenia, to the same scale, we selected a representative study that measures the change in symptoms on the PANSS scale [15]. The mean endpoint ${\mathrm{mean}}_{\mathrm{rep}.\mathrm{study}}$ and standard deviation ${\mathrm{SDpooled}}_{\mathrm{rep}.\mathrm{study}}$ for Placebo, to be used in Equations (3) and (4) of Additional file 3 to obtain the absolute mean score, were 98.4 and 21.4, respectively. Since the outcomes must be between 0 and 1, we have standardised the absolute mean score using the formula ${y}_{i}= \frac{{M}_{i}-30}{210- 30}$ (PANSS score can range between 30 and 210), as described in Equation (5) of Additional file 3. Then, the obtained value was reversed so that higher values equate to better outcomes ($1-{y}_{i}$).

The absolute probabilities ${y}_{i}$ of risk for antiparkinson medication use were obtained using Equations (1) and (2) of Additional file 3 by estimating the odds for placebo by meta-analysing the reference arms; ${y}_{\mathrm{Placebo}}$ was estimated to be 0.093 as reported in Additional file 5.

The absolute risk probabilities ${y}_{i}$ for the remaining harmful outcomes, weight gain, prolactin elevation, and QTc prolongation, were converted from the corresponding continuous outcomes using Equation (6) of Additional file 3. To derive the control group probabilities ${p}_{\mathrm{Placebo}}$ (Equation (7) of Additional file 3) we used the dichotomous variables to distinguish patients with and without the response based on a cut-off *C* of at least 7% for weight gain and study-specific thresholds for prolactin elevation and QTc prolongation. The estimated ${p}_{\mathrm{Placebo}}$ values were 0.034, 0.019, and 0.006, for weight gain, prolactin elevation, and QTc prolongation, respectively. The obtained probabilities and corresponding SMDs for each treatment are available in Additional file 5. Due to missing data for one or more outcomes, 18 antipsychotics were not included in the calculation of the ${\mathrm{SAWIS}}_{i}$.

**Fig. 2:**
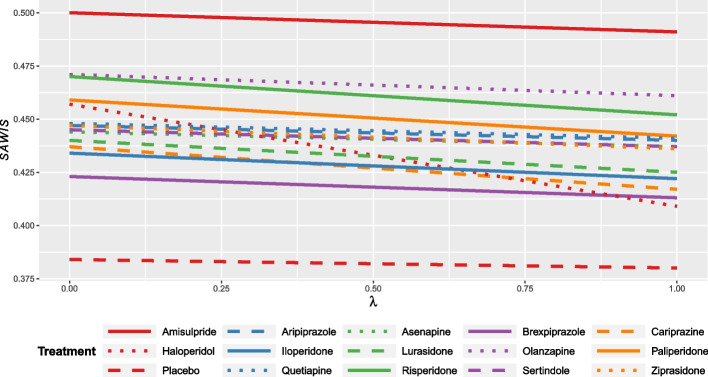
${SAWIS}_{i}$ quantity for the network of antipsychotics for the acute treatment of multi-episode schizophrenia. The benefit spie chart included only one efficacy outcome, overall symptoms of schizophrenia, and the harm spie chart included antiparkinson medication use, weight gain, prolactin elevation, and QTc prolongation with weights 0.4, 0.3, 0.15, and 0.15, respectively

After consulting with clinicians, we gave a weight of 0.4 and 0.3 to antiparkinson medication use and weight gain, respectively, to reflect the importance of these safety outcomes compared to the other two which were both given a weight of 0.15. The ${\mathrm{SAWIS}}_{i}$ values for different $\lambda$ values are shown in Additional file 6 and illustrated in Fig. 2.

The decrease of ${\mathrm{SAWIS}}_{i}$ is particularly evident for haloperidol which, for the chosen weights, goes from being among the best treatments to being the worst active drug when the willingness to tolerate harm decreases. Whatever the trade-off between benefits and harms, placebo remains the least preferable treatment, while amisulpride, olanzapine, and risperidone remain the most preferable.

### Ranking pharmacological and dietary‑supplement treatments for autism spectrum disorder 

For this example, it was not possible to estimate absolute probabilities or use any of the conversion methods described previously due to the variety of scales used to calculate this score and the lack of a specific cut-off to define responders using these scales. Therefore, for all outcomes, we first estimated the relative treatment effects (SMD for continuous outcomes and OR for the safety outcome) of each intervention versus placebo and then, produced the relative SUCRA that we employed as the outcome measures ${y}_{ij}$ (Equation (1)) to plot in the spie charts. For the safety outcome, any adverse event, we calculated the SUCRA to reflect the fact that in the spie chart framework, higher values for the safety outcomes must indicate higher harm, as previously explained. Therefore, the corresponding SUCRA ranking is reversed compared to what the ordinary SUCRA ranking for a safety outcome would look like, i.e. the best-performing treatment in terms of rate of adverse events will have the lowest SUCRA value in our case, instead of the highest value, as it would usually be. The calculated SUCRA values are reported in Additional file 7.

We calculated ${SAWIS}_{i}$ by giving a weight of 0.5 to both efficacy outcomes, i.e. social-communication difficulties and repetitive behaviours. Due to missing data for one or two outcomes, 16 interventions were not included in the calculation of the ${SAWIS}_{i}$.

**Fig. 3:**
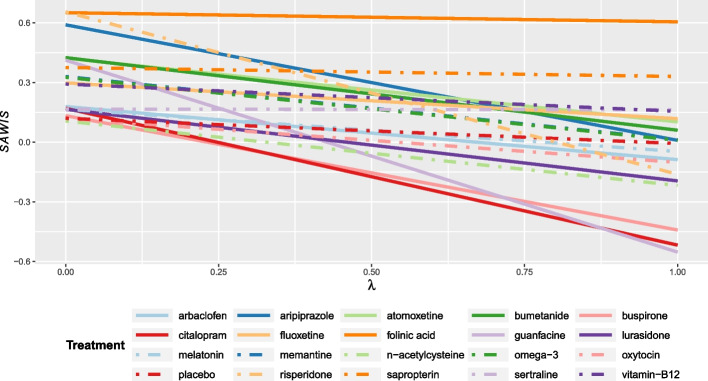
${SAWIS}_{i}$ quantity for the network of pharmacological and dietary‑supplement treatments for autism spectrum disorder. The benefit spie chart included two efficacy outcomes, changes in core symptoms for social-communication difficulties, and repetitive behaviours with weights 0.5 each, and the safety spie chart included one outcome, any adverse event

The ${SAWIS}_{i}$ values for different trade-off values are reported in Additional file 8 and illustrated in Fig. 3. The ${\mathrm{SAWIS}}_{i}$ decrease, for increasing values of $\lambda$, is nearly null for some treatments, such as folinic acid, sapropterin, and sertraline. However, for some treatments the decrease in the ${\mathrm{SAWIS}}_{i}$ values is so large that they shift from being the most efficacious treatments to being among the least beneficial ones, particularly risperidone and guanfacine.

Unlike the previous examples, the range of ${\mathrm{SAWIS}}_{i}$ for this example is quite broad, also including negative values. This is because the quantities here are calculated from SUCRA which estimates the probability that a treatment *X* outperforms its competitors *Y*, *Z*, etc., and hence depends on the performance of the competitors *Y*, *Z*, etc. Consequently, SUCRA values can be large (e.g. above 0.7) for “high-performing” interventions, even when the outcome is rare (as in safety outcomes). Therefore, the SUCRA values for harm outcomes, when included in the spie charts and, in turn, in the ${SAWIS}_{i}$ formula, could then produce negative values if the corresponding SUCRAs for positive outcomes are not of the same magnitude.

## Discussion

In this manuscript, we presented an innovative and easy-to-implement method to show if the performance of a treatment varies for different trade-offs between efficacy and safety/acceptability outcomes by combining the area within a spie chart for both benefits and harms. We tested this approach with three different datasets (and different outcomes) from network meta-analyses published in different fields of mental health; however, this approach can be generalised to other fields of medicine.

Being an extension of the spie chart approach, our method shares the same limitations. First, this is only applicable when all treatments have data available for all outcomes of interest. As the author of the original spie chart paper reports, one option, when using SUCRA as the outcome measure ${y}_{i}$, is to include the outcome in the calculation of the areas within the spie charts by assigning an outcome value of zero to those treatments where it is unavailable. In this way, however, such treatments are penalised for having missing outcomes. For this reason, in the antipsychotics and autism examples, we decided to exclude the treatments with missing outcome data, following implicitly the assumption that these are not relevant for the purpose of evaluating treatment performance. Second, the choice of outcomes to be included and their relative importance should be based on clinical grounds. Furthermore, the choice of outcomes must be given careful consideration not only in relation to the availability of data but also with regard to the correlation between the specific outcomes and the information they provide. For example, for antidepressants, we decided to exclude the continuous outcome from rating scales as we thought they would essentially duplicate the information already given by the response rate. Then, the values produced by this method depend greatly on the choice of the outcome measures to include in the spie charts which must also be on the same scale. We described in Additional file 3 how we obtained the absolute probabilities for binary outcomes and how we converted the continuous outcomes into a dichotomous scale [11–13] but reviewers should consult available guidance in the literature to obtain absolute probabilities for other types of outcomes, e.g. rate or time-to-event data [16]. Also, the absolute probabilities of response (or risk) do not account for the uncertainty of the relative treatment effects, which is instead encompassed by a measure such as the SUCRA, or the P-score, which is still between 0 and 1. We have, however, shown in the third illustrative example how the use of SUCRA affects the results in our approach, creating more variation across the range of the trade-off values. Similar conclusions are shown when using SUCRA values as outcome measures in the spie charts for the other two examples (Additional file 9). As explained in the “Results” section, this is due to the nature of the SUCRA, whose value depends on the performance of a treatment compared to all its competitors. We, therefore, recommend the use of absolute probabilities or absolute mean effects scaled between 0 and 1 over the use of SUCRA values for our approach, whenever possible.

Like any other method of presenting results from network meta-analysis, illustrating the ${SAWIS}_{i}$ values can become more difficult as the number of treatments increases. We produced plots to show the variation across the whole range of lambda, but users can choose their preferred way to visualise it.

We want to draw attention to the fact that the quantities produced by our method should not be regarded as a new ranking metric. The interpretation of the area within a spie chart is not straightforward itself as it depends on the outcome measures plotted in the charts. Additionally, the final quantity we obtain from our approach is a difference between the two coverage areas adjusted for a specified trade-off value which adds further complexity to the interpretation. Specifically, we made our trade-off equal to the ratio 1/*u*, where *u* could be set by the answer to questions such as “how much harm could you tolerate for an increase in benefit?” so that *λ* ranges between 0 and 1. However, *u* should theoretically be in the same unit as ${A}_{i}^{H}$, the value of the area within the harms spie chart, to make the final value of our method interpreted as a proper ranking metric. Future research could expand this method and focus on the interpretation of the trade-off *λ* and the produced value. Also, as the name of our approach suggests, the quantity refers to the benefit with a specific treatment adjusted for different levels of harm. An equivalent measure expressed in terms of harms could be produced similarly, e.g. the “net harm”, where harm is adjusted for different degrees of benefit. However, patients and clinicians usually think more in terms of benefits gained, hence we believe that our proposed method could be more intuitive to implement.

We recommend using our method as a sensitivity analysis to check if the performance of a treatment stays consistent when multiple outcomes and different preferences are considered. These preferences are expressed by the importance of the different outcomes, represented by the weights in the spie chart formula and by the trade-off *λ* that allows to indicate “how much” one is willing to tolerate to see an increase in benefit. As this trade-off is bound to remain very subjective, it is even more important to examine the variability for all plausible values of *λ*. This is also why a “summary measure”, e.g. averaging over all values of *λ*, would not prove very informative. The loss of granularity due to aggregating the results from different *λ* values would not make the user appreciate the variability properly.

In the broader context of comparing treatments, health economic modelling and, specifically, cost-effectiveness analysis is sometimes used on top of NMA results to assess the performance of competing treatments accounting for costs. Our proposed approach draws from the cost-effectiveness methodology and uses the net-benefit concept to trade-off between harms and benefits. However, costs cannot be considered the same as harms as they can also vary substantially by country and reimbursement systems. When the interest lies in ranking treatments according to the cost-effectiveness profile, a proper economic evaluation approach would be required.

Various visualisation tools have been proposed lately to facilitate the communication of results for multiple outcomes [17, 18]. However, unlike our new approach, they all assume the different benefits and harms outcomes have the same importance. We believe that the method described in this paper could help clinicians, patients, and policy-makers to make decisions on which treatment is preferable when multiple outcomes are of interest and trading-off between benefits and harms is important.

### Supplementary Information

**Additional file 1.** Network graphs of the three motivating examples.

**Additional file 2.** Spie charts for the included treatments and outcomes of the three motivating examples.

**Additional file 3.** Methods to transform outcome measures on a 0 to 1 scale.

**Additional file 4. **${SAWIS}_{i}$ values for different $\lambda$ for the network of 18 antidepressants.

**Additional file 5. **Absolute probabilities and corresponding SMDs for the outcomes of the network of antipsychotics.

**Additional file 6. **The ${\mathrm{SAWIS}}_{i}$ values for different $\lambda$ values for the network of antipsychotics.

**Additional file 7. **Calculated SUCRA values for otcomes of the network of treatments for autism spectrum disorder.

**Additional file 8. **The ${SAWIS}_{i}$ values for different $\lambda$ values for the network of treatments for autism spectrum disorder.

**Additional file 9. **The ${SAWIS}_{i}$ values for the networks of antipressants and antipsychotics using SUCRA values as outcome measures in the spie charts.

## Data Availability

The datasets generated and analysed, and the code to replicate the results study are available in the GitHub repository https://github.com/esm-ispm-unibe-ch/nb-spie.

## Abbreviations

NMA: Network meta-analysis
SUCRA: Surface under the cumulative ranking curve
OR: Odds ratio
MD: Mean difference
SMD: Standardised mean difference
SD: Standard deviation
SAWIS: Net-benefit standardised area within a spie chart
