# Supplementary material for: Estimating and visualising the trade-off between benefits and harms on multiple clinical outcomes in network meta-analysis
Source: Syst Rev. 2023 Nov 11;12:209. doi: 10.1186/s13643-023-02376-1 (PMC10638812; doi:10.1186/s13643-023-02376-1)

Figure A: Network of head-to-head studies investigating 18 antidepressants for the acute treatment of adults with major depressive disorder (Cipriani et al. Lancet 2018)


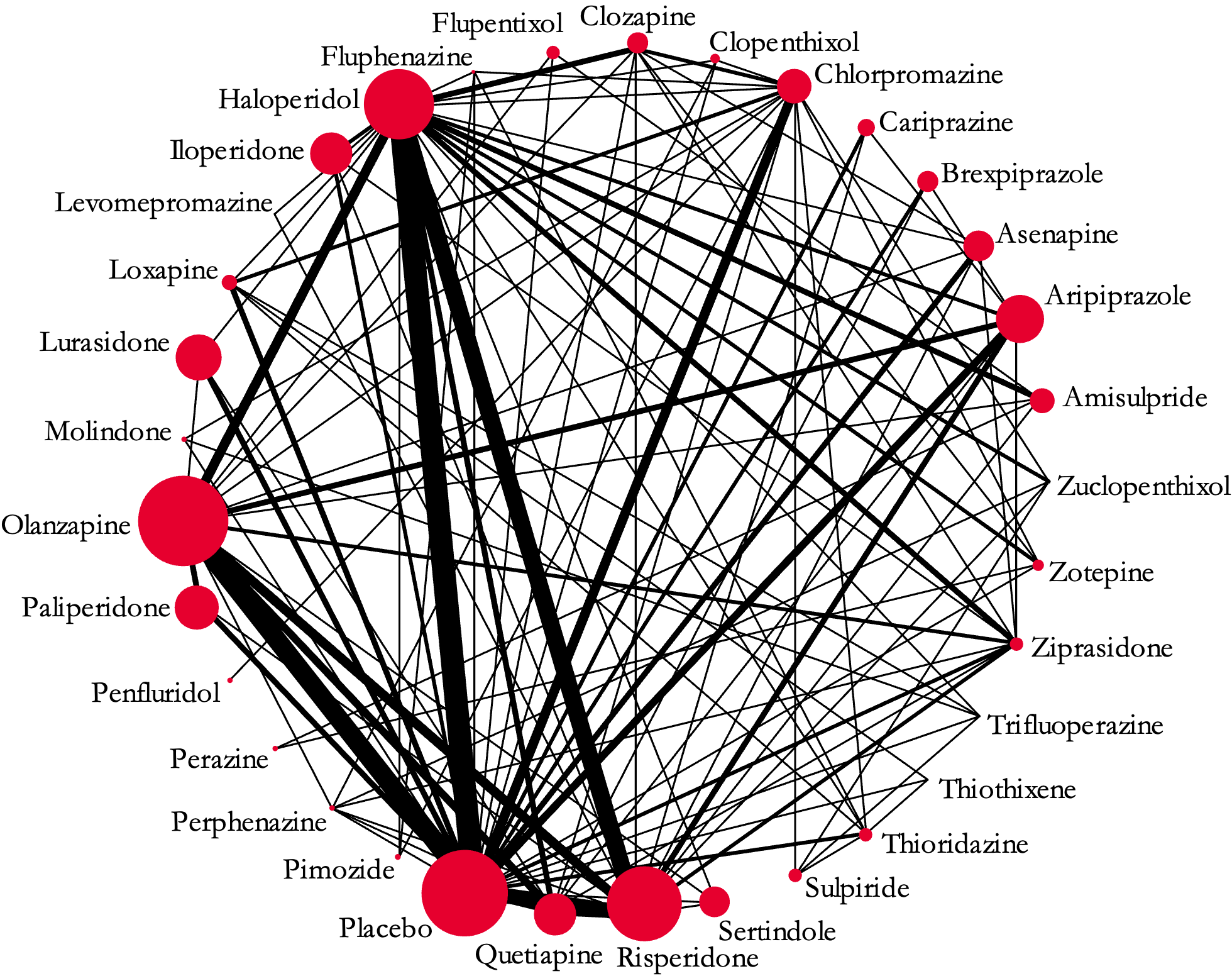


Figure B: Network of placebo-controlled studies of 32 oral antipsychotics for the acute treatment of adults with multi-episode schizophrenia (Huhn et al. Lancet 2019)

Figure C: Network of pharmacological and dietary‑supplement treatments for autism spectrum disorder (Siafis et al. Molecular Autism 2022)


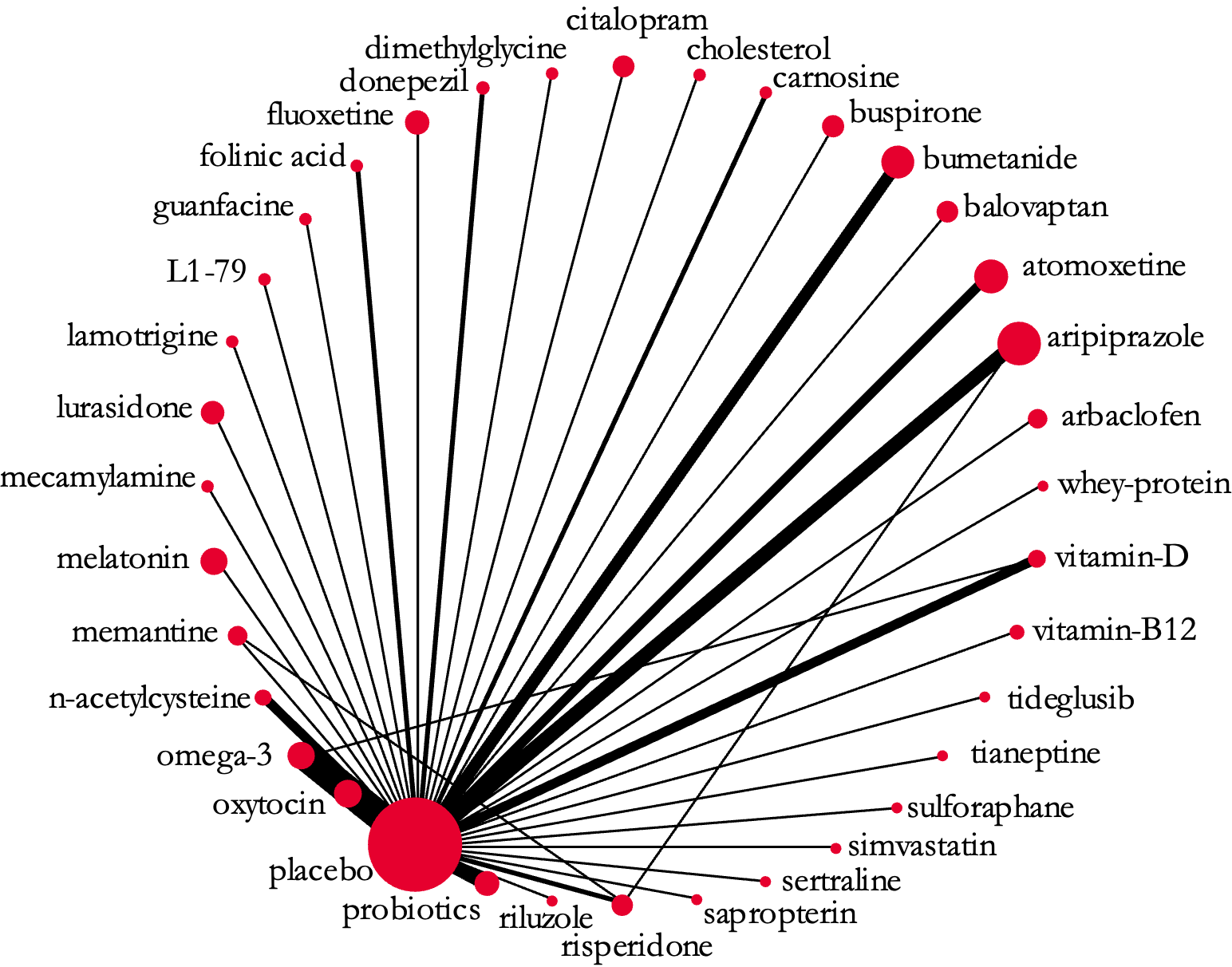

Supplement: Supplementary file 1 — Additional file 1. Network graphs of the three motivating examples. [file 13643_2023_2376_MOESM1_ESM.docx]
