# Supplementary material for: Estimating and visualising the trade-off between benefits and harms on multiple clinical outcomes in network meta-analysis
Source: Syst Rev. 2023 Nov 11;12:209. doi: 10.1186/s13643-023-02376-1 (PMC10638812; doi:10.1186/s13643-023-02376-1)

Figure A: Spie charts for the efficacy outcomes (response to treatment and remission) of the network of 18 antidepressants; agom = agomelatine, amit = amitriptyline, bupr = bupropion, cita = citalopram, clom = clomipramine, dulo = duloxetine, esci = escitalopram, fluo = fluoxetine, fluv = fluvoxamine, miln = milnacipran, mirt = mirtazapine, nefa = nefazodone, paro = paroxetine, rebo = reboxetine, sert = sertraline, traz = trazodone, venl = venlafaxine, vort = vortioxetine.


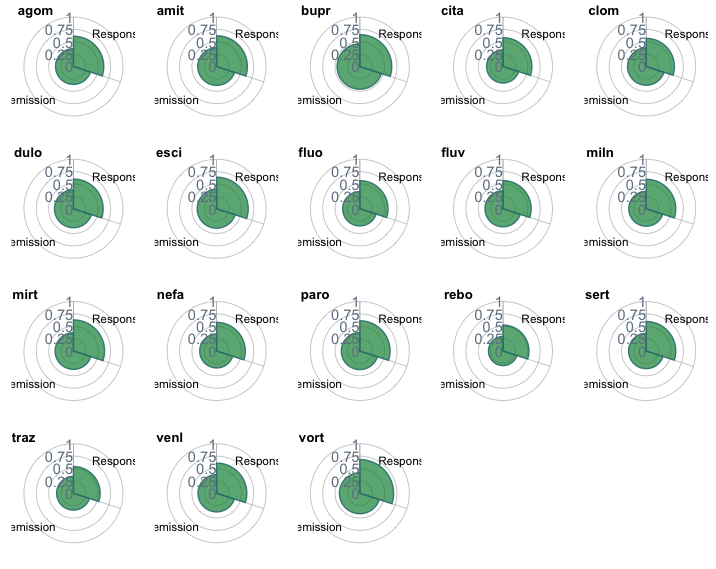


Figure B: Spie charts for the safety outcomes (dropout due to any cause and dropout due to adverse events) of the network of 18 antidepressants; agom = agomelatine, amit = amitriptyline, bupr = bupropion, cita = citalopram, clom = clomipramine, dulo = duloxetine, esci = escitalopram, fluo = fluoxetine, fluv = fluvoxamine, miln = milnacipran, mirt = mirtazapine, nefa = nefazodone, paro = paroxetine, rebo = reboxetine, sert = sertraline, traz = trazodone, venl = venlafaxine, vort = vortioxetine.


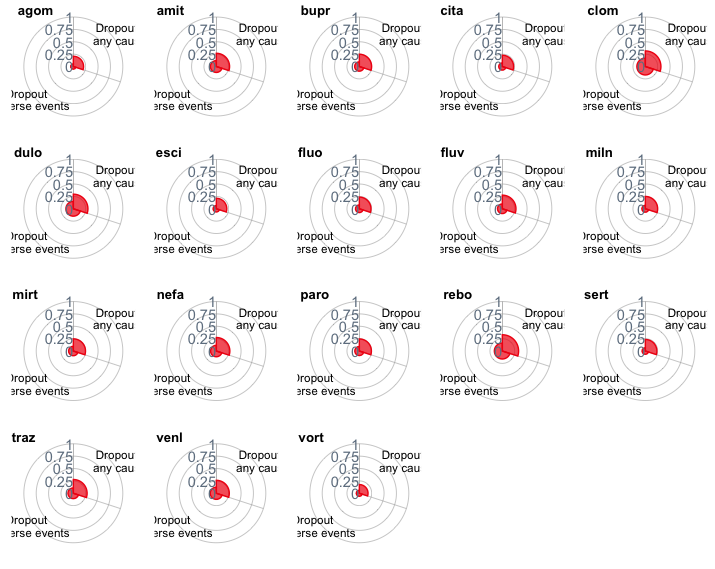


Figure C: Spie charts for the efficacy outcome (overall symptoms of schizophrenia) of the network of antipsychotics for the treatment of schizophrenia.


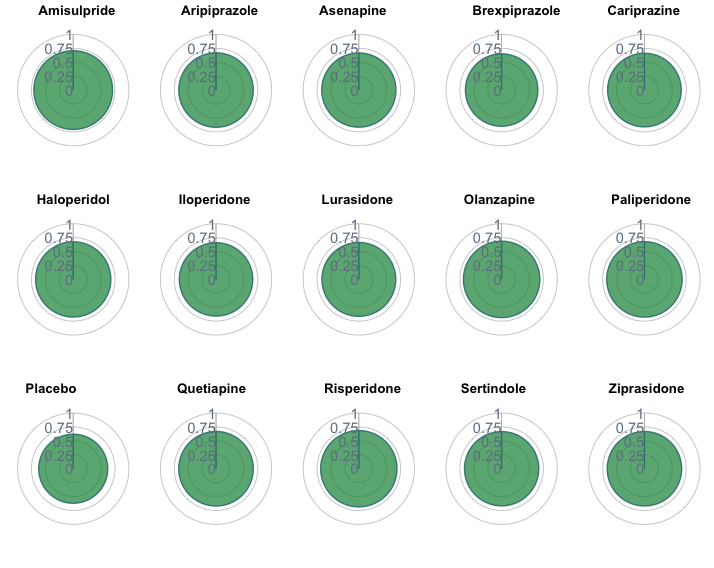


Figure D: Spie charts for the safety outcomes (use of antiparkinson medication, weight gain, prolactin elevation, and QTc prolongation) of the network of antipsychotics for the treatment of schizophrenia.


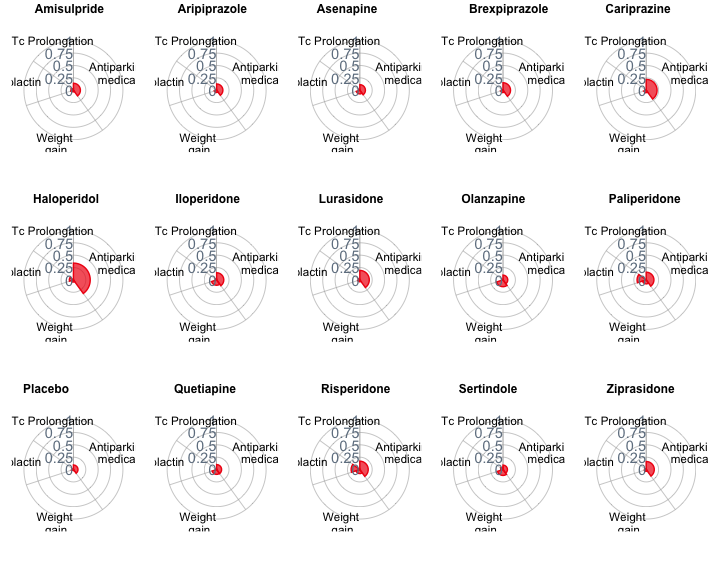


Figure E: Spie charts for the efficacy outcomes (changes in core symptoms for social-communication difficulties and repetitive behaviours) for the network of treatments for autism spectrum disorder.


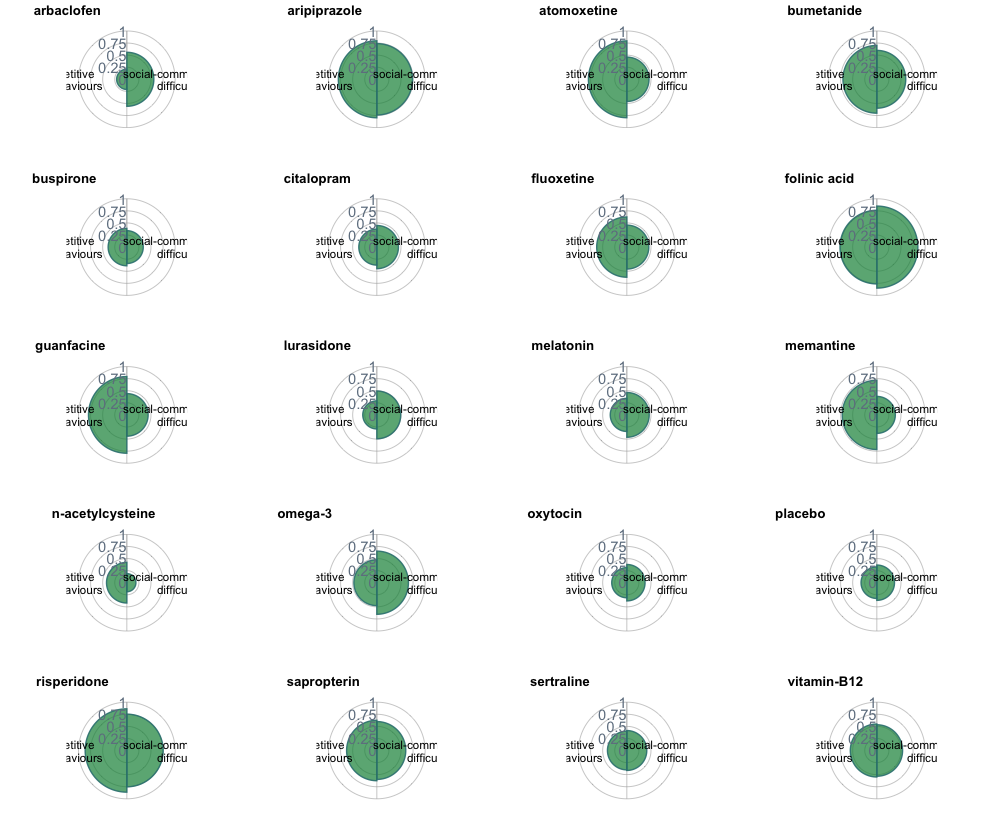


Figure F: Spie charts for the safety outcome (patients with adverse events) for the network of treatments for autism spectrum disorder.


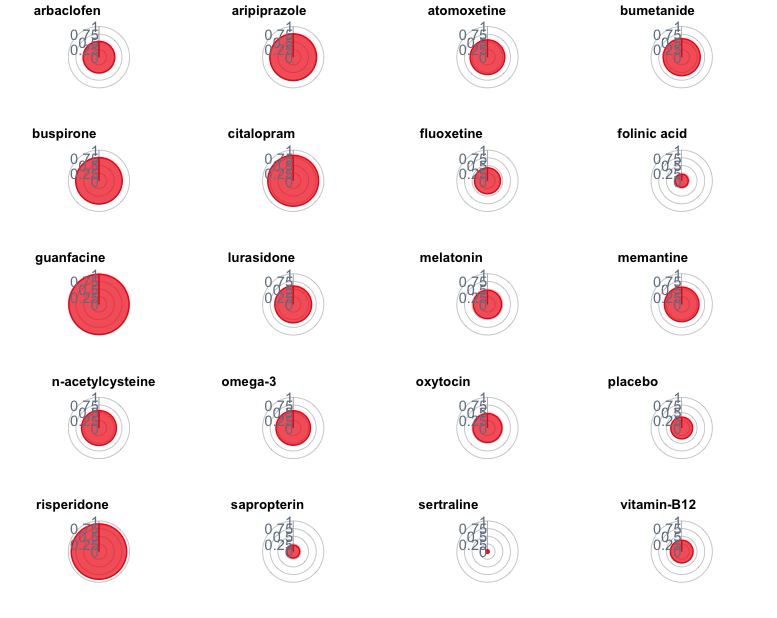

Supplement: Supplementary file 2 — Additional file 2. Spie charts for the included treatments and outcomes of the three motivating examples. [file 13643_2023_2376_MOESM2_ESM.docx]
