# Supplementary material for: Estimating and visualising the trade-off between benefits and harms on multiple clinical outcomes in network meta-analysis
Source: Syst Rev. 2023 Nov 11;12:209. doi: 10.1186/s13643-023-02376-1 (PMC10638812; doi:10.1186/s13643-023-02376-1)
Supplement: Supplementary file 3 — Additional file 3. Methods to transform outcome measures on a 0 to 1 scale. [file 13643_2023_2376_MOESM3_ESM.docx]

*Transforming outcome measures on a 0 to 1 scale: Dichotomous outcomes*

For each treatment *i* the absolute probabilities $y_{i}$ for dichotomous outcomes can be calculated by using the odds ratios ${OR}_{i}$ versus control from the outcome-specific network meta-analyses and the odds in the control group ${odds}_{control}$ which can be estimated by meta-analysing the reference arms.

$$\begin{aligned} y_{i}=\frac{{odds}_{i}}{1+{odds}_{i}} \#[1] \end{aligned}$$

$$\begin{aligned} {odds}_{i}={OR}_{i}\times{odds}_{control} \#[2] \end{aligned}$$

*Transforming outcome measures on a 0 to 1 scale: Continuous outcomes*

If the continuous outcome is measured using different scales (e.g. symptoms scores or rating scales), the mean differences ${MD}_{i}$ of each treatment *i* versus the control group can be calculated using the relative standardised mean differences ${SMD}_{i}$ versus the control group from the outcome-specific network meta-analysis and the pooled standard deviation ${SDpooled}_{rep.study}$ from a representative study in the field reporting the outcome in the chosen scale.

$$\begin{aligned} {MD}_{i}={SMD}_{i}*{SDpooled}_{rep.study} \#[3] \end{aligned}$$

The absolute mean effects $M_{i}$ for each treatment *i* can then be calculated from the ${MD}_{i}$ and the absolute mean for the control group ${mean}_{rep.study}$ from the chosen representative study

$$\begin{aligned} M_{i}={MD}_{i}+{mean}_{rep.study}\#[4] \end{aligned}$$

If the continuous outcome is measured on a scale where a defined minimum and maximum value exists, the obtained absolute mean effects $M_{i}$ for each treatment *i* are standardised using the minimum and maximum values, $min$ and $max$, for the relevant outcome scale

$$\begin{aligned} y_{i}= \frac{M_{i}-min}{\max- min}\#[5] \end{aligned}$$

If the continuous outcome is not defined within a specific range, it can be converted into a "response/risk" probabilities $y_{i}$ from the control group probability $p_{control}$ and the ${SMD}_{i}$ of group *i* versus the control group using Furukawa's method (da Costa et al, Furukawa et al, Furukawa & Leucht)

$$\begin{aligned} y_{i}=\Phi\left( {SMD}_{i}-\Phi^{-1}\left( 1-p_{control} \right) \right)\#[6] \end{aligned}$$

where $\Phi$ is the cumulative standard normal distribution and $\Phi^{-1}$ its inverse. The control group probabilities $p_{control}$ represents the probability of scores of patients beyond the cut-off value *C*, used to distinguish between those with and without treatment response for that continuous outcome, and calculated from the mean ${mean}_{control}$ and standard deviation ${SD}_{control}$ in the control group

$$\begin{aligned} p_{control}=\Phi\left( \frac{{mean}_{control}-C}{{SD}_{control}} \right)\#[7] \end{aligned}$$

If dichotomous variables defining how many patients reach the specific cut-off *C* are available, $p_{control}$ can also be estimated from a meta-analysis of proportions.

Another way to transform outcomes measured on the same scale is with the use of a *partial value function* as described by Tervonen et al. The idea is to bound the region in which the outcome values are likely to fall by setting two points $c_{k}^{'}$ and $c_{k}^{''}$as the least and most preferable values, respectively. A (linear) partial value function could then be defined as $u_{k}\left( c_{k} \right)=(c_{k}-c_{k}^{'})/(c_{k}^{''}-c_{k}^{'})$, for an outcome where larger values are preferable, and it is normalized by $u_{k}\left( c_{k}^{'} \right)=0$ and $u_{k}\left( c_{k}^{'} \right)=1$.
