# Supplementary material for: Estimating and visualising the trade-off between benefits and harms on multiple clinical outcomes in network meta-analysis
Source: Syst Rev. 2023 Nov 11;12:209. doi: 10.1186/s13643-023-02376-1 (PMC10638812; doi:10.1186/s13643-023-02376-1)
Supplement: Supplementary file 4 — Additional file 4. ${SAWIS}_{i}$SAWISi values for different $\lambda$λ for the network of 18 antidepressants. [file 13643_2023_2376_MOESM4_ESM.docx]

| λ | agom | amit | bupr | cita | clom | dulo | esci | fluo | fluv | miln | mirt | nefa | paro | rebo | sert | traz | venl | vort |
| --- | --- | --- | --- | --- | --- | --- | --- | --- | --- | --- | --- | --- | --- | --- | --- | --- | --- | --- |
| 0 | 0.204 | 0.217 | 0.272 | 0.18 | 0.197 | 0.211 | 0.23 | 0.181 | 0.19 | 0.194 | 0.215 | 0.182 | 0.21 | 0.143 | 0.196 | 0.169 | 0.211 | 0.259 |
| 0.05 | 0.20325 | 0.2154 | 0.27075 | 0.17905 | 0.1945 | 0.2089 | 0.22915 | 0.17995 | 0.1885 | 0.1929 | 0.21375 | 0.1804 | 0.2088 | 0.14045 | 0.195 | 0.1674 | 0.20945 | 0.2584 |
| 0.1 | 0.2025 | 0.2138 | 0.2695 | 0.1781 | 0.192 | 0.2068 | 0.2283 | 0.1789 | 0.187 | 0.1918 | 0.2125 | 0.1788 | 0.2076 | 0.1379 | 0.194 | 0.1658 | 0.2079 | 0.2578 |
| 0.15 | 0.20175 | 0.2122 | 0.26825 | 0.17715 | 0.1895 | 0.2047 | 0.22745 | 0.17785 | 0.1855 | 0.1907 | 0.21125 | 0.1772 | 0.2064 | 0.13535 | 0.193 | 0.1642 | 0.20635 | 0.2572 |
| 0.2 | 0.201 | 0.2106 | 0.267 | 0.1762 | 0.187 | 0.2026 | 0.2266 | 0.1768 | 0.184 | 0.1896 | 0.21 | 0.1756 | 0.2052 | 0.1328 | 0.192 | 0.1626 | 0.2048 | 0.2566 |
| 0.25 | 0.20025 | 0.209 | 0.26575 | 0.17525 | 0.1845 | 0.2005 | 0.22575 | 0.17575 | 0.1825 | 0.1885 | 0.20875 | 0.174 | 0.204 | 0.13025 | 0.191 | 0.161 | 0.20325 | 0.256 |
| 0.3 | 0.1995 | 0.2074 | 0.2645 | 0.1743 | 0.182 | 0.1984 | 0.2249 | 0.1747 | 0.181 | 0.1874 | 0.2075 | 0.1724 | 0.2028 | 0.1277 | 0.19 | 0.1594 | 0.2017 | 0.2554 |
| 0.35 | 0.19875 | 0.2058 | 0.26325 | 0.17335 | 0.1795 | 0.1963 | 0.22405 | 0.17365 | 0.1795 | 0.1863 | 0.20625 | 0.1708 | 0.2016 | 0.12515 | 0.189 | 0.1578 | 0.20015 | 0.2548 |
| 0.4 | 0.198 | 0.2042 | 0.262 | 0.1724 | 0.177 | 0.1942 | 0.2232 | 0.1726 | 0.178 | 0.1852 | 0.205 | 0.1692 | 0.2004 | 0.1226 | 0.188 | 0.1562 | 0.1986 | 0.2542 |
| 0.45 | 0.19725 | 0.2026 | 0.26075 | 0.17145 | 0.1745 | 0.1921 | 0.22235 | 0.17155 | 0.1765 | 0.1841 | 0.20375 | 0.1676 | 0.1992 | 0.12005 | 0.187 | 0.1546 | 0.19705 | 0.2536 |
| 0.5 | 0.1965 | 0.201 | 0.2595 | 0.1705 | 0.172 | 0.19 | 0.2215 | 0.1705 | 0.175 | 0.183 | 0.2025 | 0.166 | 0.198 | 0.1175 | 0.186 | 0.153 | 0.1955 | 0.253 |
| 0.55 | 0.19575 | 0.1994 | 0.25825 | 0.16955 | 0.1695 | 0.1879 | 0.22065 | 0.16945 | 0.1735 | 0.1819 | 0.20125 | 0.1644 | 0.1968 | 0.11495 | 0.185 | 0.1514 | 0.19395 | 0.2524 |
| 0.6 | 0.195 | 0.1978 | 0.257 | 0.1686 | 0.167 | 0.1858 | 0.2198 | 0.1684 | 0.172 | 0.1808 | 0.2 | 0.1628 | 0.1956 | 0.1124 | 0.184 | 0.1498 | 0.1924 | 0.2518 |
| 0.65 | 0.19425 | 0.1962 | 0.25575 | 0.16765 | 0.1645 | 0.1837 | 0.21895 | 0.16735 | 0.1705 | 0.1797 | 0.19875 | 0.1612 | 0.1944 | 0.10985 | 0.183 | 0.1482 | 0.19085 | 0.2512 |
| 0.7 | 0.1935 | 0.1946 | 0.2545 | 0.1667 | 0.162 | 0.1816 | 0.2181 | 0.1663 | 0.169 | 0.1786 | 0.1975 | 0.1596 | 0.1932 | 0.1073 | 0.182 | 0.1466 | 0.1893 | 0.2506 |
| 0.75 | 0.19275 | 0.193 | 0.25325 | 0.16575 | 0.1595 | 0.1795 | 0.21725 | 0.16525 | 0.1675 | 0.1775 | 0.19625 | 0.158 | 0.192 | 0.10475 | 0.181 | 0.145 | 0.18775 | 0.25 |
| 0.8 | 0.192 | 0.1914 | 0.252 | 0.1648 | 0.157 | 0.1774 | 0.2164 | 0.1642 | 0.166 | 0.1764 | 0.195 | 0.1564 | 0.1908 | 0.1022 | 0.18 | 0.1434 | 0.1862 | 0.2494 |
| 0.85 | 0.19125 | 0.1898 | 0.25075 | 0.16385 | 0.1545 | 0.1753 | 0.21555 | 0.16315 | 0.1645 | 0.1753 | 0.19375 | 0.1548 | 0.1896 | 0.09965 | 0.179 | 0.1418 | 0.18465 | 0.2488 |
| 0.9 | 0.1905 | 0.1882 | 0.2495 | 0.1629 | 0.152 | 0.1732 | 0.2147 | 0.1621 | 0.163 | 0.1742 | 0.1925 | 0.1532 | 0.1884 | 0.0971 | 0.178 | 0.1402 | 0.1831 | 0.2482 |
| 0.95 | 0.18975 | 0.1866 | 0.24825 | 0.16195 | 0.1495 | 0.1711 | 0.21385 | 0.16105 | 0.1615 | 0.1731 | 0.19125 | 0.1516 | 0.1872 | 0.09455 | 0.177 | 0.1386 | 0.18155 | 0.2476 |
| 1 | 0.189 | 0.185 | 0.247 | 0.161 | 0.147 | 0.169 | 0.213 | 0.16 | 0.16 | 0.172 | 0.19 | 0.15 | 0.186 | 0.092 | 0.176 | 0.137 | 0.18 | 0.247 |
| agom = agomelatine, amit = amitriptyline, bupr = bupropion, cita = citalopram, clom = clomipramine, dulo = duloxetine, esci = escitalopram, fluo = fluoxetine, fluv = fluvoxamine, miln = milnacipran, mirt = mirtazapine, nefa = nefazodone, paro = paroxetine, rebo = reboxetine, sert = sertraline, traz = trazodone, venl = venlafaxine, vort = vortioxetine. | | | | | | | | | | | | | | | | | | |
