# Supplementary material for: Estimating and visualising the trade-off between benefits and harms on multiple clinical outcomes in network meta-analysis
Source: Syst Rev. 2023 Nov 11;12:209. doi: 10.1186/s13643-023-02376-1 (PMC10638812; doi:10.1186/s13643-023-02376-1)
Supplement: Supplementary file 5 — Additional file 5. Absolute probabilities and corresponding SMDs for the outcomes of the network of antipsychotics. [file 13643_2023_2376_MOESM5_ESM.docx]

|  | id | Prob efficacy | Prob antiparkinson | Prob weight gain | SMD weight | Prob prolactin | SMD prolactin | Prob QTc Prolongation | SMD qtc |
| --- | --- | --- | --- | --- | --- | --- | --- | --- | --- |
| Amisulpride | 1 | 0.707 | 0.141 | 0.036 | 0.031 | 0.055 | 0.480 | 0.038 | 0.759 |
| Aripiprazole | 2 | 0.669 | 0.127 | 0.050 | 0.180 | 0.011 | -0.203 | 0.006 | 0.000 |
| Asenapine | 3 | 0.666 | 0.113 | 0.076 | 0.392 | 0.028 | 0.168 | 0.012 | 0.269 |
| Brexpiprazole | 4 | 0.650 | 0.152 | 0.051 | 0.194 | 0.026 | 0.134 | 0.004 | -0.086 |
| Cariprazine | 5 | 0.661 | 0.219 | 0.054 | 0.217 | 0.015 | -0.102 | 0.005 | -0.048 |
| Chlorpromazine | 6 | 0.673 | 0.212 | 0.125 | 0.678 | 0.026 | 0.127 | #N/A | #N/A |
| Clopenthixol | 7 | 0.669 | 0.516 | 0.032 | -0.021 | #N/A | #N/A | #N/A | #N/A |
| Clozapine | 8 | 0.726 | 0.041 | 0.131 | 0.704 | #N/A | #N/A | #N/A | #N/A |
| Flupentixol | 9 | 0.671 | 0.331 | 0.043 | 0.114 | 0.016 | -0.057 | #N/A | #N/A |
| Fluphenazine | 10 | 0.650 | 0.404 | #N/A | #N/A | #N/A | #N/A | #N/A | #N/A |
| Haloperidol | 11 | 0.676 | 0.340 | 0.047 | 0.156 | 0.084 | 0.698 | 0.007 | 0.099 |
| Iloperidone | 12 | 0.659 | 0.145 | 0.109 | 0.594 | 0.029 | 0.185 | 0.014 | 0.337 |
| Levomepromazine | 13 | 0.623 | 0.131 | 0.118 | 0.642 | #N/A | #N/A | #N/A | #N/A |
| Loxapine | 14 | 0.673 | 0.324 | 0.067 | 0.331 | #N/A | #N/A | #N/A | #N/A |
| Lurasidone | 15 | 0.663 | 0.192 | 0.041 | 0.087 | 0.037 | 0.287 | 0.004 | -0.104 |
| Molindone | 16 | 0.669 | 0.297 | 0.008 | -0.586 | #N/A | #N/A | #N/A | #N/A |
| Olanzapine | 17 | 0.686 | 0.097 | 0.138 | 0.738 | 0.027 | 0.147 | 0.011 | 0.228 |
| Paliperidone | 18 | 0.678 | 0.156 | 0.082 | 0.434 | 0.186 | 1.182 | 0.007 | 0.049 |
| Penfluridol | 19 | 0.667 | 0.344 | #N/A | #N/A | #N/A | #N/A | #N/A | #N/A |
| Perazine | 20 | 0.655 | 0.068 | 0.030 | -0.054 | 0.004 | -0.592 | #N/A | #N/A |
| Perphenazine | 21 | 0.687 | 0.263 | #N/A | #N/A | #N/A | #N/A | #N/A | #N/A |
| Pimozide | 22 | 0.656 | 0.804 | #N/A | #N/A | 0.024 | 0.095 | #N/A | #N/A |
| Placebo | 23 | 0.620 | 0.093 | 0.034 | 0.000 | 0.019 | 0.000 | 0.006 | 0.000 |
| Quetiapine | 24 | 0.669 | 0.101 | 0.098 | 0.534 | 0.015 | -0.084 | 0.009 | 0.162 |
| Risperidone | 25 | 0.685 | 0.170 | 0.083 | 0.439 | 0.177 | 1.148 | 0.011 | 0.225 |
| Sertindole | 26 | 0.667 | 0.090 | 0.119 | 0.649 | 0.052 | 0.451 | 0.053 | 0.917 |
| Sulpiride | 27 | 0.677 | 0.249 | 0.025 | -0.126 | #N/A | #N/A | #N/A | #N/A |
| Thioridazine | 28 | 0.685 | 0.103 | #N/A | #N/A | #N/A | #N/A | #N/A | #N/A |
| Thiothixene | 29 | 0.695 | 0.420 | #N/A | #N/A | #N/A | #N/A | #N/A | #N/A |
| Trifluoperazine | 30 | 0.649 | 0.288 | 0.026 | -0.112 | #N/A | #N/A | #N/A | #N/A |
| Ziprasidone | 31 | 0.669 | 0.163 | 0.039 | 0.063 | 0.028 | 0.167 | 0.017 | 0.412 |
| Zotepine | 32 | 0.692 | 0.198 | 0.170 | 0.873 | 0.000 | -1.782 | #N/A | #N/A |
| Zuclopenthixol | 33 | 0.681 | 0.286 | 0.100 | 0.544 | #N/A | #N/A | #N/A | #N/A |
