# Supplementary material for: Estimating and visualising the trade-off between benefits and harms on multiple clinical outcomes in network meta-analysis
Source: Syst Rev. 2023 Nov 11;12:209. doi: 10.1186/s13643-023-02376-1 (PMC10638812; doi:10.1186/s13643-023-02376-1)
Supplement: Supplementary file 6 — Additional file 6. The ${\mathrm{SAWIS}}_{i}$SAWISi values for different $\lambda$λ values for the network of antipsychotics. [file 13643_2023_2376_MOESM6_ESM.docx]

| **λ** | **Amisulpride** | **Aripiprazole** | **Asenapine** | **Brexpiprazole** | **Cariprazine** | **Haloperidol** | **Iloperidone** | **Lurasidone** | **Olanzapine** | **Paliperidone** | **Placebo** | **Quetiapine** | **Risperidone** | **Sertindole** | **Ziprasidone** |
| --- | --- | --- | --- | --- | --- | --- | --- | --- | --- | --- | --- | --- | --- | --- | --- |
| **0** | 0.5 | 0.447 | 0.444 | 0.423 | 0.437 | 0.457 | 0.434 | 0.44 | 0.471 | 0.459 | 0.384 | 0.448 | 0.47 | 0.445 | 0.447 |
| **0.05** | 0.49955 | 0.44665 | 0.44365 | 0.4225 | 0.436 | 0.4546 | 0.4334 | 0.43925 | 0.4705 | 0.45815 | 0.3838 | 0.44765 | 0.4691 | 0.4446 | 0.44645 |
| **0.1** | 0.4991 | 0.4463 | 0.4433 | 0.422 | 0.435 | 0.4522 | 0.4328 | 0.4385 | 0.47 | 0.4573 | 0.3836 | 0.4473 | 0.4682 | 0.4442 | 0.4459 |
| **0.15** | 0.49865 | 0.44595 | 0.44295 | 0.4215 | 0.434 | 0.4498 | 0.4322 | 0.43775 | 0.4695 | 0.45645 | 0.3834 | 0.44695 | 0.4673 | 0.4438 | 0.44535 |
| **0.2** | 0.4982 | 0.4456 | 0.4426 | 0.421 | 0.433 | 0.4474 | 0.4316 | 0.437 | 0.469 | 0.4556 | 0.3832 | 0.4466 | 0.4664 | 0.4434 | 0.4448 |
| **0.25** | 0.49775 | 0.44525 | 0.44225 | 0.4205 | 0.432 | 0.445 | 0.431 | 0.43625 | 0.4685 | 0.45475 | 0.383 | 0.44625 | 0.4655 | 0.443 | 0.44425 |
| **0.3** | 0.4973 | 0.4449 | 0.4419 | 0.42 | 0.431 | 0.4426 | 0.4304 | 0.4355 | 0.468 | 0.4539 | 0.3828 | 0.4459 | 0.4646 | 0.4426 | 0.4437 |
| **0.35** | 0.49685 | 0.44455 | 0.44155 | 0.4195 | 0.43 | 0.4402 | 0.4298 | 0.43475 | 0.4675 | 0.45305 | 0.3826 | 0.44555 | 0.4637 | 0.4422 | 0.44315 |
| **0.4** | 0.4964 | 0.4442 | 0.4412 | 0.419 | 0.429 | 0.4378 | 0.4292 | 0.434 | 0.467 | 0.4522 | 0.3824 | 0.4452 | 0.4628 | 0.4418 | 0.4426 |
| **0.45** | 0.49595 | 0.44385 | 0.44085 | 0.4185 | 0.428 | 0.4354 | 0.4286 | 0.43325 | 0.4665 | 0.45135 | 0.3822 | 0.44485 | 0.4619 | 0.4414 | 0.44205 |
| **0.5** | 0.4955 | 0.4435 | 0.4405 | 0.418 | 0.427 | 0.433 | 0.428 | 0.4325 | 0.466 | 0.4505 | 0.382 | 0.4445 | 0.461 | 0.441 | 0.4415 |
| **0.55** | 0.49505 | 0.44315 | 0.44015 | 0.4175 | 0.426 | 0.4306 | 0.4274 | 0.43175 | 0.4655 | 0.44965 | 0.3818 | 0.44415 | 0.4601 | 0.4406 | 0.44095 |
| **0.6** | 0.4946 | 0.4428 | 0.4398 | 0.417 | 0.425 | 0.4282 | 0.4268 | 0.431 | 0.465 | 0.4488 | 0.3816 | 0.4438 | 0.4592 | 0.4402 | 0.4404 |
| **0.65** | 0.49415 | 0.44245 | 0.43945 | 0.4165 | 0.424 | 0.4258 | 0.4262 | 0.43025 | 0.4645 | 0.44795 | 0.3814 | 0.44345 | 0.4583 | 0.4398 | 0.43985 |
| **0.7** | 0.4937 | 0.4421 | 0.4391 | 0.416 | 0.423 | 0.4234 | 0.4256 | 0.4295 | 0.464 | 0.4471 | 0.3812 | 0.4431 | 0.4574 | 0.4394 | 0.4393 |
| **0.75** | 0.49325 | 0.44175 | 0.43875 | 0.4155 | 0.422 | 0.421 | 0.425 | 0.42875 | 0.4635 | 0.44625 | 0.381 | 0.44275 | 0.4565 | 0.439 | 0.43875 |
| **0.8** | 0.4928 | 0.4414 | 0.4384 | 0.415 | 0.421 | 0.4186 | 0.4244 | 0.428 | 0.463 | 0.4454 | 0.3808 | 0.4424 | 0.4556 | 0.4386 | 0.4382 |
| **0.85** | 0.49235 | 0.44105 | 0.43805 | 0.4145 | 0.42 | 0.4162 | 0.4238 | 0.42725 | 0.4625 | 0.44455 | 0.3806 | 0.44205 | 0.4547 | 0.4382 | 0.43765 |
| **0.9** | 0.4919 | 0.4407 | 0.4377 | 0.414 | 0.419 | 0.4138 | 0.4232 | 0.4265 | 0.462 | 0.4437 | 0.3804 | 0.4417 | 0.4538 | 0.4378 | 0.4371 |
| **0.95** | 0.49145 | 0.44035 | 0.43735 | 0.4135 | 0.418 | 0.4114 | 0.4226 | 0.42575 | 0.4615 | 0.44285 | 0.3802 | 0.44135 | 0.4529 | 0.4374 | 0.43655 |
| **1** | 0.491 | 0.44 | 0.437 | 0.413 | 0.417 | 0.409 | 0.422 | 0.425 | 0.461 | 0.442 | 0.38 | 0.441 | 0.452 | 0.437 | 0.436 |
