# Supplementary material for: Estimating and visualising the trade-off between benefits and harms on multiple clinical outcomes in network meta-analysis
Source: Syst Rev. 2023 Nov 11;12:209. doi: 10.1186/s13643-023-02376-1 (PMC10638812; doi:10.1186/s13643-023-02376-1)
Supplement: Supplementary file 7 — Additional file 7. Calculated SUCRA values for otcomes of the network of treatments for autism spectrum disorder. [file 13643_2023_2376_MOESM7_ESM.docx]

|  | **id** | **social_SUCRA** | **repbehav_SUCRA** | **ae_SUCRA** |
| --- | --- | --- | --- | --- |
| **arbaclofen** | 1 | 0.5591 | 0.2111 | 0.5171 |
| **aripiprazole** | 2 | 0.7387 | 0.7966 | 0.7625 |
| **atomoxetine** | 3 | 0.4593 | 0.7951 | 0.5657 |
| **balovaptan** | 4 | 0.3452 | #N/A | 0.4071 |
| **bumetanide** | 5 | 0.5984 | 0.7014 | 0.6043 |
| **buspirone** | 6 | 0.3401 | 0.3870 | 0.7586 |
| **carnosine** | 7 | 0.4933 | 0.3512 | #N/A |
| **cholesterol** | 8 | 0.6743 | #N/A | #N/A |
| **citalopram** | 9 | 0.4472 | 0.3731 | 0.8296 |
| **dimethylglycine** | 10 | 0.8216 | #N/A | 0.2075 |
| **donepezil** | 11 | 0.2807 | #N/A | #N/A |
| **fluoxetine** | 12 | 0.4549 | 0.6236 | 0.4239 |
| **folinic acid** | 13 | 0.8491 | 0.7624 | 0.2148 |
| **guanfacine** | 14 | 0.4408 | 0.7937 | 0.9825 |
| **L1-79** | 15 | 0.6872 | 0.8622 | #N/A |
| **lamotrigine** | 16 | 0.3145 | 0.3591 | #N/A |
| **lurasidone** | 17 | 0.4942 | 0.2912 | 0.5999 |
| **mecamylamine** | 18 | 0.1853 | 0.3729 | #N/A |
| **melatonin** | 19 | 0.4632 | 0.3431 | 0.4617 |
| **memantine** | 20 | 0.3828 | 0.7163 | 0.5642 |
| **n-acetylcysteine** | 21 | 0.1884 | 0.4205 | 0.5692 |
| **omega-3** | 22 | 0.6539 | 0.4747 | 0.5626 |
| **oxytocin** | 23 | 0.3773 | 0.3126 | 0.4710 |
| **placebo** | 24 | 0.3660 | 0.3269 | 0.3557 |
| **probiotics** | 25 | 0.6682 | 0.3031 | #N/A |
| **riluzole** | 26 | 0.5580 | #N/A | 0.0960 |
| **risperidone** | 27 | 0.7519 | 0.8624 | 0.9041 |
| **sapropterin** | 28 | 0.5988 | 0.6256 | 0.2125 |
| **sertraline** | 29 | 0.4131 | 0.4012 | 0.0589 |
| **simvastatin** | 30 | 0.1946 | 0.1861 | #N/A |
| **sulforaphane** | 31 | 0.2934 | 0.3938 | #N/A |
| **tianeptine** | 32 | 0.5555 | #N/A | #N/A |
| **tideglusib** | 33 | 0.7778 | 0.6461 | #N/A |
| **vitamin-B12** | 34 | 0.5330 | 0.5486 | 0.3706 |
| **vitamin-D** | 35 | 0.4229 | 0.2583 | #N/A |
| **whey-protein** | 36 | 0.6174 | #N/A | #N/A |
