# Supplementary material for: Estimating and visualising the trade-off between benefits and harms on multiple clinical outcomes in network meta-analysis
Source: Syst Rev. 2023 Nov 11;12:209. doi: 10.1186/s13643-023-02376-1 (PMC10638812; doi:10.1186/s13643-023-02376-1)
Supplement: Supplementary file 8 — Additional file 8. The ${SAWIS}_{i}$SAWISi values for different $\lambda$λ values for the network of treatments for autism spectrum disorder. [file 13643_2023_2376_MOESM8_ESM.docx]

| **λ** | **arbaclofen** | **aripiprazole** | **atomoxetine** | **bumetanide** | **buspirone** | **citalopram** | **fluoxetine** | **folinic acid** | **guanfacine** | **lurasidone** | **melatonin** | **memantine** | **n-acetylcysteine** | **omega3** | **oxytocin** | **placebo** | **risperidone** | **sapropterin** | **sertraline** | **vitamin B12** |
| --- | --- | --- | --- | --- | --- | --- | --- | --- | --- | --- | --- | --- | --- | --- | --- | --- | --- | --- | --- | --- |
| **0** | 0.179 | 0.590 | 0.422 | 0.425 | 0.133 | 0.170 | 0.298 | 0.651 | 0.412 | 0.165 | 0.166 | 0.330 | 0.106 | 0.326 | 0.120 | 0.120 | 0.655 | 0.375 | 0.166 | 0.292 |
| **0.05** | 0.166 | 0.561 | 0.406 | 0.407 | 0.104 | 0.136 | 0.289 | 0.649 | 0.364 | 0.147 | 0.155 | 0.314 | 0.090 | 0.310 | 0.109 | 0.114 | 0.614 | 0.373 | 0.166 | 0.285 |
| **0.1** | 0.152 | 0.532 | 0.390 | 0.389 | 0.076 | 0.101 | 0.280 | 0.646 | 0.316 | 0.129 | 0.145 | 0.298 | 0.074 | 0.294 | 0.098 | 0.107 | 0.573 | 0.371 | 0.166 | 0.278 |
| **0.15** | 0.139 | 0.503 | 0.374 | 0.370 | 0.047 | 0.067 | 0.271 | 0.644 | 0.267 | 0.111 | 0.134 | 0.282 | 0.057 | 0.278 | 0.087 | 0.101 | 0.532 | 0.368 | 0.166 | 0.271 |
| **0.2** | 0.126 | 0.474 | 0.358 | 0.352 | 0.018 | 0.032 | 0.262 | 0.642 | 0.219 | 0.093 | 0.123 | 0.266 | 0.041 | 0.263 | 0.076 | 0.095 | 0.492 | 0.366 | 0.165 | 0.265 |
| **0.25** | 0.112 | 0.445 | 0.342 | 0.334 | -0.011 | -0.002 | 0.253 | 0.640 | 0.171 | 0.075 | 0.113 | 0.251 | 0.025 | 0.247 | 0.065 | 0.088 | 0.451 | 0.364 | 0.165 | 0.258 |
| **0.3** | 0.099 | 0.416 | 0.326 | 0.316 | -0.040 | -0.036 | 0.244 | 0.637 | 0.123 | 0.057 | 0.102 | 0.235 | 0.009 | 0.231 | 0.053 | 0.082 | 0.410 | 0.362 | 0.165 | 0.251 |
| **0.35** | 0.086 | 0.387 | 0.310 | 0.297 | -0.068 | -0.071 | 0.235 | 0.635 | 0.074 | 0.039 | 0.091 | 0.219 | -0.007 | 0.215 | 0.042 | 0.076 | 0.369 | 0.359 | 0.165 | 0.244 |
| **0.4** | 0.072 | 0.358 | 0.294 | 0.279 | -0.097 | -0.105 | 0.226 | 0.633 | 0.026 | 0.021 | 0.081 | 0.203 | -0.024 | 0.199 | 0.031 | 0.069 | 0.328 | 0.357 | 0.165 | 0.237 |
| **0.45** | 0.059 | 0.329 | 0.278 | 0.261 | -0.126 | -0.140 | 0.217 | 0.630 | -0.022 | 0.003 | 0.070 | 0.187 | -0.040 | 0.183 | 0.020 | 0.063 | 0.287 | 0.355 | 0.165 | 0.230 |
| **0.5** | 0.046 | 0.300 | 0.262 | 0.243 | -0.155 | -0.174 | 0.208 | 0.628 | -0.071 | -0.015 | 0.060 | 0.171 | -0.056 | 0.168 | 0.009 | 0.057 | 0.247 | 0.353 | 0.165 | 0.224 |
| **0.55** | 0.032 | 0.270 | 0.246 | 0.224 | -0.183 | -0.208 | 0.199 | 0.626 | -0.119 | -0.033 | 0.049 | 0.155 | -0.072 | 0.152 | -0.002 | 0.050 | 0.206 | 0.350 | 0.164 | 0.217 |
| **0.6** | 0.019 | 0.241 | 0.230 | 0.206 | -0.212 | -0.243 | 0.190 | 0.623 | -0.167 | -0.051 | 0.038 | 0.139 | -0.088 | 0.136 | -0.013 | 0.044 | 0.165 | 0.348 | 0.164 | 0.210 |
| **0.65** | 0.005 | 0.212 | 0.214 | 0.188 | -0.241 | -0.277 | 0.181 | 0.621 | -0.215 | -0.069 | 0.028 | 0.123 | -0.105 | 0.120 | -0.024 | 0.037 | 0.124 | 0.346 | 0.164 | 0.203 |
| **0.7** | -0.008 | 0.183 | 0.198 | 0.170 | -0.270 | -0.312 | 0.172 | 0.619 | -0.264 | -0.087 | 0.017 | 0.107 | -0.121 | 0.104 | -0.035 | 0.031 | 0.083 | 0.344 | 0.164 | 0.196 |
| **0.75** | -0.021 | 0.154 | 0.182 | 0.151 | -0.298 | -0.346 | 0.163 | 0.617 | -0.312 | -0.105 | 0.006 | 0.092 | -0.137 | 0.088 | -0.047 | 0.025 | 0.042 | 0.341 | 0.164 | 0.189 |
| **0.8** | -0.035 | 0.125 | 0.166 | 0.133 | -0.327 | -0.380 | 0.154 | 0.614 | -0.360 | -0.123 | -0.004 | 0.076 | -0.153 | 0.072 | -0.058 | 0.018 | 0.001 | 0.339 | 0.164 | 0.182 |
| **0.85** | -0.048 | 0.096 | 0.150 | 0.115 | -0.356 | -0.415 | 0.145 | 0.612 | -0.408 | -0.141 | -0.015 | 0.060 | -0.169 | 0.057 | -0.069 | 0.012 | -0.039 | 0.337 | 0.163 | 0.176 |
| **0.9** | -0.061 | 0.067 | 0.134 | 0.097 | -0.385 | -0.449 | 0.136 | 0.610 | -0.457 | -0.159 | -0.026 | 0.044 | -0.186 | 0.041 | -0.080 | 0.006 | -0.080 | 0.335 | 0.163 | 0.169 |
| **0.95** | -0.075 | 0.038 | 0.118 | 0.078 | -0.413 | -0.484 | 0.127 | 0.607 | -0.505 | -0.177 | -0.036 | 0.028 | -0.202 | 0.025 | -0.091 | -0.001 | -0.121 | 0.332 | 0.163 | 0.162 |
| **1** | -0.088 | 0.009 | 0.102 | 0.060 | -0.442 | -0.518 | 0.118 | 0.605 | -0.553 | -0.195 | -0.047 | 0.012 | -0.218 | 0.009 | -0.102 | -0.007 | -0.162 | 0.330 | 0.163 | 0.155 |
