# Supplementary material for: Estimating and visualising the trade-off between benefits and harms on multiple clinical outcomes in network meta-analysis
Source: Syst Rev. 2023 Nov 11;12:209. doi: 10.1186/s13643-023-02376-1 (PMC10638812; doi:10.1186/s13643-023-02376-1)
Supplement: Supplementary file 9 [file 13643_2023_2376_MOESM9_ESM.docx]

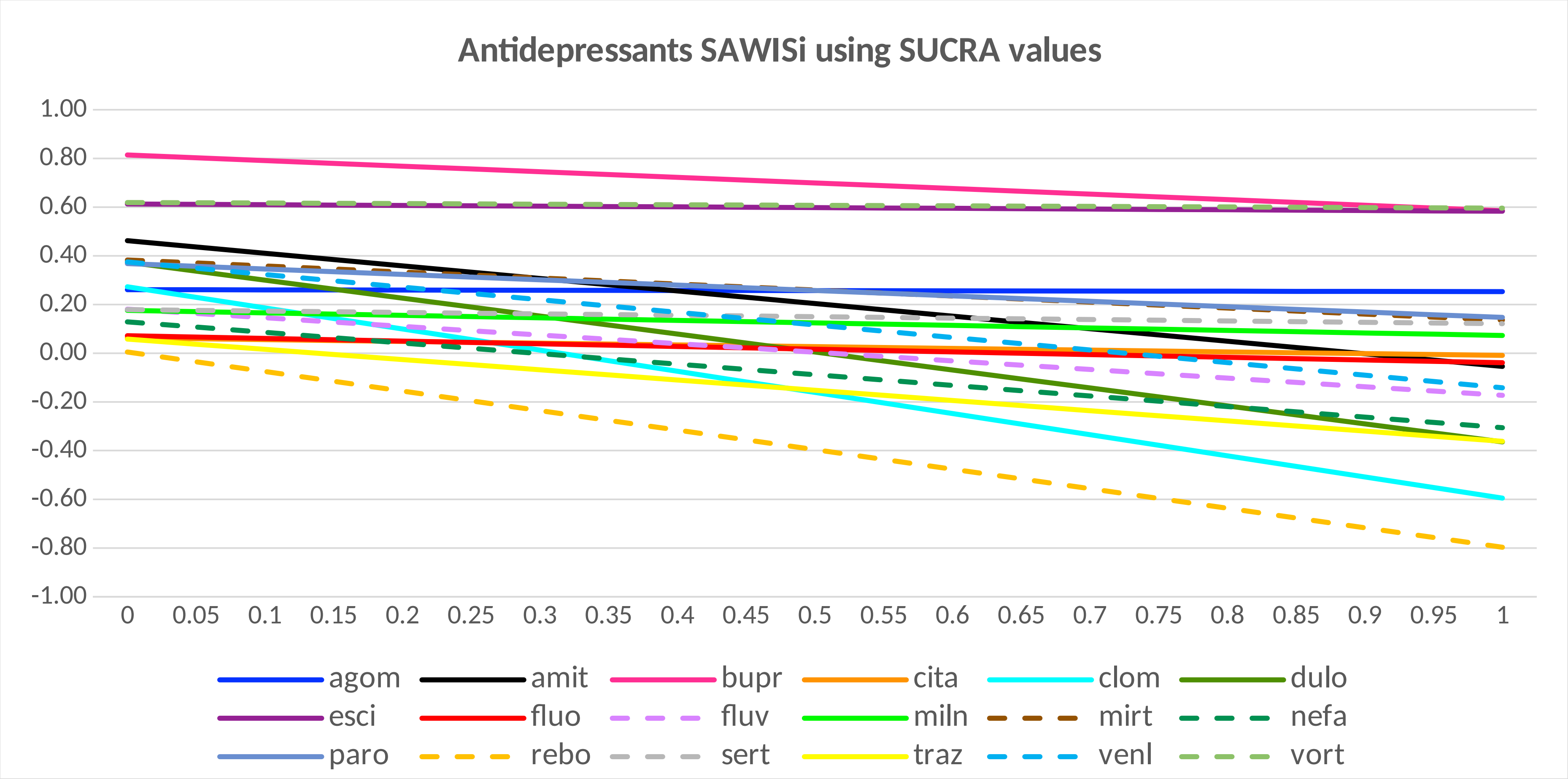
Antidepressants SAWISi using SUCRA values
1.00
0.80
0.60
0.40
0.20
0.00
-0.20
-0.40
-0.60
-0.80
-1.00
0
0.05
0.1
0.15
0.2
0.25
0.3
0.35
0.4
0.45
0.5
0.55
0.6
0.65
0.7
0.75
0.8
0.85
0.9
0.95
1
agom
amit
bupr
cita
clom
dulo
esci
fluo
fluv
miln
mirt
nefa
paro
rebo
sert
traz
venl
vort

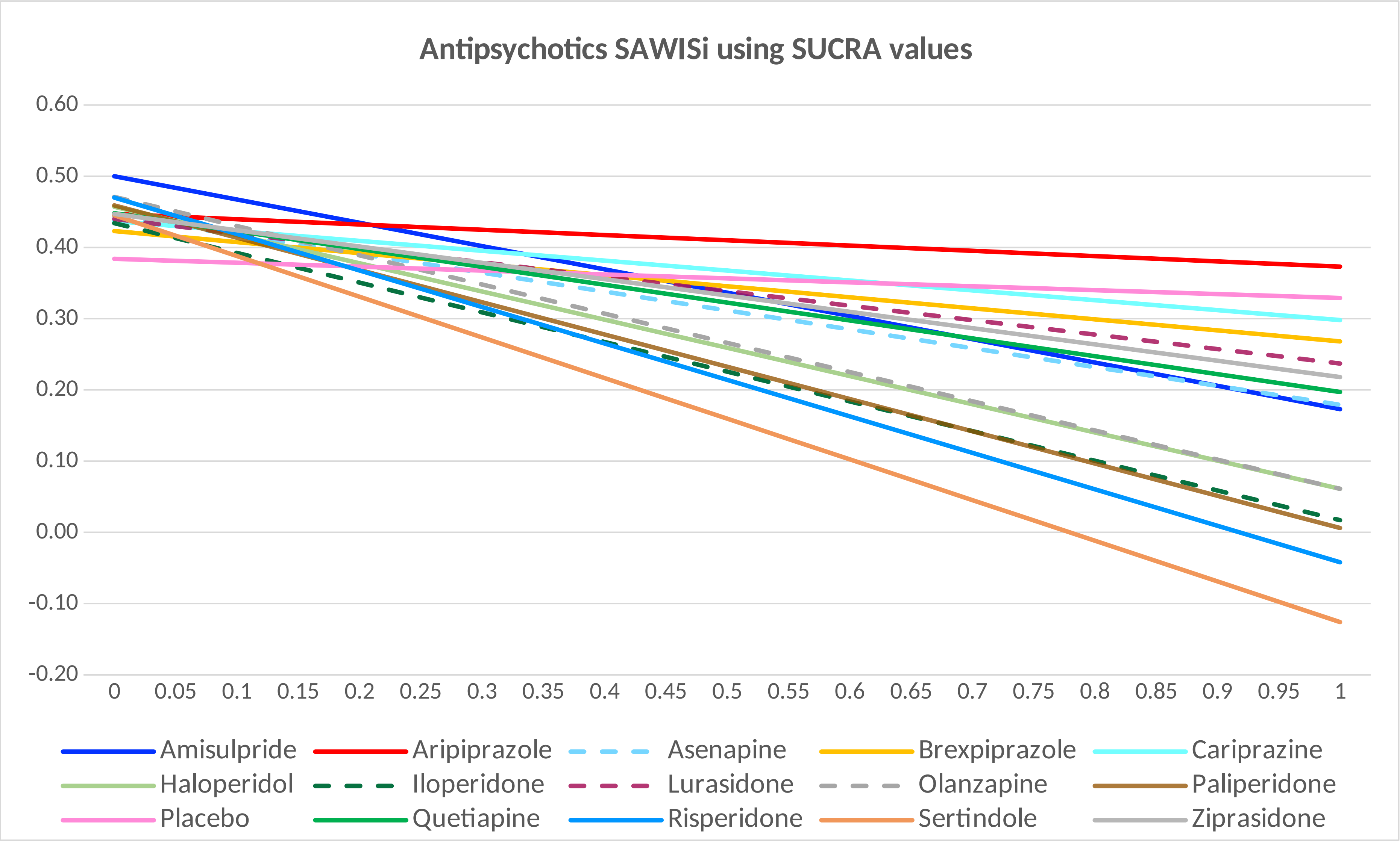

Antipsychotics SAWISi using SUCRA values
0.60
0.50
0.40
0.30
0.20
0.10
0.00
-0.10
-0.20
0
0.05
0.1
0.15
0.2
0.25
0.3
0.35
0.4
0.45
0.5
0.55
0.6
0.65
0.7
0.75
0.8
0.85
0.9
0.95
1
Amisulpride
Aripiprazole
Asenapine
Brexpiprazole
Cariprazine
Haloperidol
Iloperidone
Lurasidone
Olanzapine
Paliperidone
Placebo
Quetiapine
Risperidone
Sertindole
Ziprasidone
